# Pyroptotic and non‐pyroptotic effector functions of caspase‐11

**DOI:** 10.1111/imr.12910

**Published:** 2020-08-01

**Authors:** Arwa Abu Khweek, Amal O. Amer

**Affiliations:** ^1^ Department of Biology and Biochemistry Birzeit University West Bank Palestine; ^2^ Department of Microbial Infection and Immunity Infectious Disease Institute College of Medicine The Ohio State University Columbus OH USA

**Keywords:** *Burkholderia*, caspase‐1, caspase‐11, danger‐associated molecular patterns, gasdermin, *Legionella*, pathogen‐associated molecular patterns, pyroptosis, *Salmonella*

## Abstract

Innate immune cells, epithelial cells, and many other cell types are capable of detecting infection or tissue injury, thus mounting regulated immune response. Inflammasomes are highly sophisticated and effective orchestrators of innate immunity. These oligomerized multiprotein complexes are at the center of various innate immune pathways, including modulation of the cytoskeleton, production and maturation of cytokines, and control of bacterial growth and cell death. Inflammasome assembly often results in caspase‐1 activation, which is an inflammatory caspase that is involved in pyroptotic cell death and release of inflammatory cytokines in response to pathogen patterns and endogenous danger stimuli. However, the nature of stimuli and inflammasome components are diverse. Caspase‐1 activation mediated release of mature IL‐1β and IL‐18 in response to canonical stimuli initiated by NOD‐like receptor (NLR), and apoptosis‐associated speck‐like protein containing a caspase recruitment domain (ASC). On the other hand, caspase‐11 delineates a non‐canonical inflammasome that promotes pyroptotic cell death and non‐pyroptotic functions in response to non‐canonical stimuli. Caspase‐11 in mice and its homologues in humans (caspase‐4/5) belong to caspase‐1 family of cysteine proteases, and play a role in inflammation. Knockout mice provided new genetic tools to study inflammatory caspases and revealed the role of caspase‐11 in mediating septic shock in response to lethal doses of lipopolysaccharide (LPS). Recognition of LPS mediates caspase‐11 activation, which promotes a myriad of downstream effects that include pyroptotic and non‐pyroptotic effector functions. Therefore, the physiological functions of caspase‐11 are much broader than its previously established roles in apoptosis and cytokine maturation. Inflammation induced by exogenous or endogenous agents can be detrimental and, if excessive, can result in organ and tissue damage. Consequently, the existence of sophisticated mechanisms that tightly regulate the specificity and sensitivity of inflammasome pathways provides a fine‐tuning balance between adequate immune response and minimal tissue damage. In this review, we summarize effector functions of caspase‐11.

## 1 INTRODUCTION

Inflammasomes assemble when a subset of intracellular receptors that belongs to the NOD‐like receptor (NLR) protein family, such as NLRP3 or NLRC4, sense PAMPs or DAMPs. Pattern sensing is followed by nucleation and oligomerization of the adapter protein ASC (apoptosis‐associated speck‐like protein containing CARD), and engagement of the cysteine protease pro‐caspase‐1.^1^, ^2^, ^3^ Within the oligomerized inflammasome complex, dimers of pro‐caspase‐1 undergo proteolytic auto‐cleavage into the enzymatically active caspase‐1, which consequently catalyzes the final processing of the inflammatory cytokines, pro‐IL‐1β and pro‐IL‐18 precursors, into their mature and secreted forms.^4^ In addition, inflammasome assembly and activation results in pyroptosis, a distinguished programmed cell death that is initiated in response to bacterial infections. It is associated with the release of inflammatory cytokines (IL‐1β, IL‐18), alarmins such as IL‐1α and high mobility group box 1 (HMGB1), and unbound and trapped bacteria within cellular debris of pyroptotic cells.^2^, ^5^, ^6^, ^7^

Assembly and activation of inflammasomes is triggered by a wide array of stimuli such as nucleic acids, bacterial toxins, and flagellin.^8^, ^9^, ^10^ Further, inflammasome activation can be mediated via endogenous damage signals such as ATP and uric acid.^11^ Activation of caspase‐1 by NLRP3/ASC or NLRC4/ASC delineates the canonical inflammasome pathway. However, the non‐canonical inflammasome requires caspase‐11‐mediated activity.

Caspase‐11 in mice, or caspase‐4/5 in humans, belongs to the family of inflammatory caspases and exhibits (46%) similarities to caspase‐1.^12^, ^13^, ^14^, ^15^, ^16^, ^17^, ^18^, ^19^ Inflammatory caspases are located on chromosome 9 in mice, and are thought to create an inflammatory cluster due to close proximity and high degree of similarity. Indeed, caspase‐11 is located adjacent to caspase‐1, only 0.012 centimorgans, or ~1500 bp, on chromosome 9 in mice.^20^ Early on, Wang et al showed that caspase‐11 induced a pyroptotic cell death in vitro and formed heterocomplex with caspase‐1, but inefficiently cleaved IL‐1β, suggesting that cytokine maturation might not solely be dependent on caspase‐11.^18^, ^21^ Generation of *Casp1^−/−^* ^22^, ^23^ and *Casp11^−/−^* ^18^ knockout mice provided the scientific community with genetic tools to study inflammatory caspases. For over a decade, it was thought that the functions of caspase‐1 and caspase‐11 are redundant as both knockout mice were resistant to lethal doses of LPS‐mediated septic shock.^18^ However, it was shown that the original *Casp1^−/−^* are double knockout and, indeed, lacked both caspase‐1 and caspase‐11.^20^ The same study showed that *Casp1^−/−^* harbors a spontaneous 5‐bp deletion in the exon 7 splice acceptor site of caspase‐11, which generated a stop codon, thus creating highly unstable caspase‐11 transcripts.^20^ To compensate for the loss of caspase‐11, *Casp1^−/−^* mice were engineered to express caspase‐11 via an artificial chromosome (*Casp1^−/−^ Casp11^Tg^*).^20^ This study showed that caspase‐11, rather than caspase‐1, mediates LPS‐induced septic shock in mice, suggesting that the functions of each caspase are distinct.^20^ The identification that *caspase1^−/−^* mice are lacking both caspase‐1 and caspase‐11^20^ promoted further research to dissect the roles of caspase‐11. We now know that the physiological function of caspase‐11 is much broader than its previously established roles in apoptosis and cytokine maturation. Herein, we discuss induction and activation and review pyroptotic and non‐pyroptotic functions of caspase‐11 that are required to control pathogens. We further discuss the role of caspase‐11 in diseases including asthma and gout. Understanding all the facets of caspase‐11 functions will help us decipher its role and contribution to inflammatory diseases and infection. It will greatly enhance our understanding to its complete role in immunity and pave the way for development of new strategies that will enhance physiological roles of caspase‐11 during infection and inflammatory diseases.

Until recently, appreciated functions of CASP11 were the recognition of cytosolic LPS followed by the activation of CASP1, cleavage of gasdermin D (GSDMD), pro‐inflammatory cytokine secretion, and cell death.

## 2 STIMULI THAT INDUCE CASPASE‐11 EXPRESSION AND ACTIVATION

Caspase‐11 is expressed broadly in immune and non‐immune cells, and its expression in resting cells is low.^18^, ^20^ In contrast to other caspases that are regulated by proteolytic cleavage, caspase‐11 is regulated at both transcriptional and post‐translational levels. Genetic analysis revealed that the caspase‐11 promoter region contains several putative binding sites for transcriptional factors including nuclear factor kappa‐light‐chain enhancer of activated B cells (NF‐κB), signal transducer and activator of transcription‐1 (STAT‐1), interferon regulatory factor (IRF), nuclear factor of activated T cells (NFAT), and cAMP response element‐binding protein (CREB).^24^ Further, induction of caspase‐11 expression is achieved by activation of p38 mitogen‐activated protein kinase (MAPK) in rat glial cells, c‐Jun N‐terminal kinase (JNK) in mouse embryonic fibroblasts, and C/EBP homologous (CHOP) protein in mice.^7^, ^25^, ^26^ Caspase‐11 expression is induced through LPS‐activated TLR4 signaling via the adapter TIR‐domain‐containing adapter‐inducing interferon‐β (TRIF) and TRIF‐dependent type I interferon (IFN) production.^6^, ^21^, ^24^, ^27^ Physiologically, during Gram‐negative bacterial infection, caspase‐11 expression and other inflammasome components are initiated concertedly by multiple TLRs in response to bacterial PAMPs. Induction of caspase‐11 and other inflammasome component expression is considered a “priming step,” and therefore is not sufficient for caspase‐11‐mediated activation and downstream effector functions. It was found that the “triggering step,” which is initiated by cytosolic recognition of LPS by caspase‐11, mediates activation and downstream effector functions of caspase‐11.^2^, ^28^ It was shown that caspase‐11 senses cholera toxin B (CTB) and extracellular bacteria such as *Escherichia coli*, *Citrobacter rodentium* and *Vibrio cholera*.^20^ Subsequent studies showed that caspase‐11 is activated by other Gram‐negative bacterial infection.^20^, ^27^, ^29^, ^30^, ^31^ We have reported the induction of caspase‐11 expression in mouse macrophages following infection with* Legionella pneumophila*. Caspase‐11 expression was shown to be independent of bacterial flagellin, ASC and the NLRC4 inflammasome.^30^ However, caspase‐11 interaction with the Nlrc4 inflammasome members and its activation required bacterial flagellin.^30^ Hence, non‐canonical stimuli such as cholera toxin or intracellular LPS trigger caspase‐11‐dependent inflammasome activation in the cytoplasm independently of TLR4.^1^, ^20^, ^32^

Recently, we have demonstrated that expression of caspase‐11 is induced by IL‐1β and IL‐1α via the IL‐1R/MYD88.^33^ Indeed, single *myd88^−/−^, trif^−/−^,* and *myd88/ trif^−/−^,* macrophages,^34^ showed reduced caspase‐11 expression in response to IL‐1α and IL‐1β, or in combination, respectively.^33^ Additionally, we have revealed that induction of caspase‐11 by LPS and HMGB1 is independent of IL‐1R. HMGB1 utilizes a variety of receptors to promote its signaling events, and LPS is sensed by TLR4, which uses both adapters to signal.^35^, ^36^ We have shown that caspase‐11 expression was not significantly hampered in the WT or the single knockout macrophages during stimulation with either HMGB1 or LPS, except when both MYD88 and TRIF were absent, indicating that either adapter was able to countervail for the deficient one in order to induce caspase‐11.^33^ Certain diseases have been classified as an IL‐1β‐mediated condition such as gout since neutralizing antibodies to IL‐1β or the caspase‐1 inhibitor z‐YVAD significantly reduce inflammation and the production of other cytokines within the joints.^37^, ^38^, ^39^ Induction of caspase‐11 expression by released IL‐1β and IL‐1α could prime immune cells for inflammasome activation by subsequent insults including monosodium urate (MSU). Thus, preventing the expression of caspase‐11 in gout‐prone individuals may prevent the instigation of tissue damage.^33^ Additionally, we have shown that house dust mites (HDM) induce caspase‐11 expression in bone marrow‐derived macrophages and in C57BL/6 mice (Abu Khweek et al under review). Intriguingly, HDM contain LPS, proteases, and chitin from the mite exoskeleton.^40^, ^41^ Therefore, the expression of caspase‐11 is induced not only by microbes but also by specific cytokines and foreign particles such as house dust mites.

## 3 CASPASE‐11 PROTEASE ACTIVITY AND SUBSTRATE SPECIFICITY

The signaling pathways upstream and downstream of caspase‐11 are still unclear, the molecular mechanism by which caspase‐11 acquires protease function in response to LPS binding or cytosolic bacterial infection is not fully understood. LPS interaction with the caspase‐11 CARD domain facilitates activation of the protease domain.^42^ Caspase‐11 is composed of an N‐terminal recruitment domain and large and small subunits that are linked by linkers. The CARD domain linker (CDL) connects the large subunit with the N‐terminal CARD domain, while the interdomain linker (IDL) connects the large and small catalytic subunits. Caspase‐11 can be detected by immunoblot into full‐length (43 kDa), shorter (38 kDa), which is thought to arise from alternative start codon within the CARD domain,^43^ and a shorter fragment generated during non‐canonical signaling in macrophages.^20^, ^44^ The catalytic cysteine C254 is located within the large subunit. Dimerization of caspase‐11 is sufficient for caspase‐11 to acquire basal proteolytic activity. However, full spectrum of caspase‐11 activities such as cleavage of gasdermin D, macrophage death, NLRP3 inflammasome activation, and IL‐1β release requires dimerization of caspase‐11 and auto‐cleavage.^45^ Indeed, caspase‐11 harbors multiple candidate sites within CDL and IDL for auto‐cleavage, which could generate fragments of multiple sizes. Ross et al showed that full‐length, uncleaved p36 and p32 cleavage fragments are released in the cell culture media in response to LPS transfection.^45^ Auto‐cleavage within IDL at residue D285, but not CDL, generates fully active P32/P10 species corresponding to CARD‐large/small subunit of caspase‐11 dimer. The generated subunits are independent of NLRP3 or caspase‐1 activation, which further implies the auto‐cleavage of caspase‐11. Human caspase‐4 is similarly cleaved to generate a p32 fragment upon exposure to cytosolic LPS or Gram‐negative bacteria, suggesting that the caspase‐11 signaling mechanism is conserved during non‐canonical inflammasome signaling in humans.^46^ Further, it has been shown that D285 residue within IDL is important for caspase‐11 function in vivo.^47^ In this study, the authors proposed that the active species of caspase‐11 are likely to be P26/P10 rather than P32/P10, wherein large subunits contains P26 generated by cleavage within the D285 and D59 or alternatively, by cleaving p36, a short form of caspase‐11 derived from an alternative start site that is lacking most of the CARD domain required for interacting with LPS. However, the precise identity of the large subunit cleavage fragment is not resolved yet. Ross et al suggest that the P32/P10, rather than P26/P10, fragments are more likely to be the cleaved fragments for several reasons. Importantly, the p36 fragment is unable to bind LPS^42^ and is unexpected to dimerize and auto‐cleave at IDL to generate p26/p10. In addition, the cleavage fragments proposed by Lee et al are unlikely to be the targets for cleavage as caspases prefer to cleave in flexible loop regions.^45^

**Figure 1:**
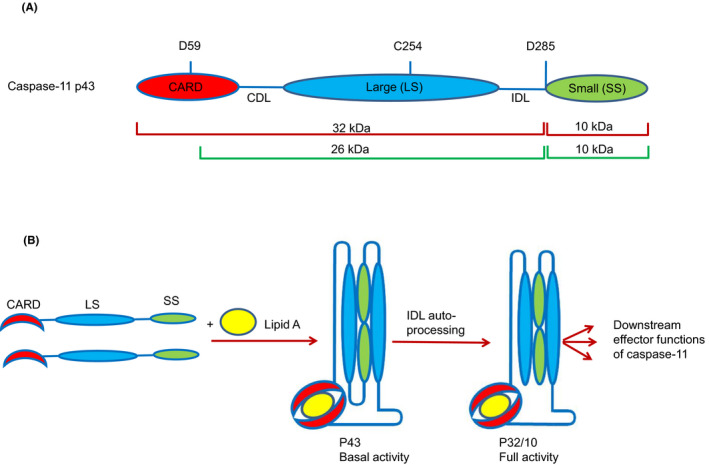
A, Domain structure of caspase‐11 showing caspase cleavage sites, the CDL, IDL, and the catalytic cysteine (C254), and the molecular weights of caspase‐11 fragments. Cleavage fragments P32/10 are shown in red lines as indicated by.^45^ Fragments p26/10 shown in green lines as demonstrated in Ref.^47^ B, Model for LPS‐induced caspase‐11 dimerization, auto‐processing, and activation as shown by Ref.^45^ Auto‐cleavage with the IDL‐ but not CDL‐mediated caspase‐11 activation and downstream effector functions including gasdermin D cleavage

In contrast to caspase‐1, caspase‐11 requires both dimerization and auto‐cleavage to mediate gasdermin D cleavage, which may or may not be associated with cell death depending on the intensity of the stimulus. The authors propose that dimerized P43 subunits of caspase‐11 have intrinsic catalytic activity, but could potentially be suboptimal to cleave substrates.^45^ Further, the dimer form could be unstable and cleavage within the IDL could potentially induce stability of the active site and substrate binding pocket.^48^, ^49^ Moreover, auto‐cleavage at IDL site may expose recognition sites for interaction with particular protein substrates, thus altering substrate specificity. Therefore, identification of caspase‐11 substrates that can be processed by dimerized but uncleaved caspase‐11 would further enhance our understanding of the function of this protease. The mechanisms regulating activation of caspase‐4/5/11 protease functions within the non‐canonical inflammasome are suggested to follow a distinct mechanism from that of caspase‐1 for several reasons. The first reason is associated with the ability of caspase‐11 to interact directly with LPS without the requirement for traditional receptor or signaling adapter.^42^ Second, non‐canonical inflammasome assembly is proposed to generate oligomers rather than dimers associated with the canonical inflammasome.^50^ Figure 1 depicts current model for caspase‐11 cleavage and activity in response to LPS.

Substrate specificity of caspase‐1 and caspase‐11 was analyzed by using massive hybrid combinatorial substrate library (HyCoSuL) screens using purified caspase‐1 and caspase‐11. The substrate preference was correlated with caspase‐11 activity on three endogenous substrates (IL‐1β, IL‐18, and gasdermin D). Caspase‐1 rapidly processed IL‐1β and IL‐18, but caspase‐11 poorly cleaved these cytokines. However, both caspase‐1 and caspase‐11 efficiently cleaved gasdermin D. The authors hypothesized that caspase‐11 might exhibit an exosite that is specifically proficient in pyroptosis, but not cleaving IL‐1β and IL‐18. Exosites are secondary binding sites that are remote from the active site and tend to direct proteases toward specific substrates that are not normally cleaved. Caspase‐11 may represent such a case by cleaving peptides and pro‐interleukins poorly, but cleaving gasdermin D well, and thus developed highly selective substrate specificity via a yet‐to‐be‐identified exosite.^51^ Therefore, caspase‐11 has restricted substrate specificity preferring gasdermin D over all the substrates examined.^20^, ^51^ Genetic evidence shows that caspase‐1 but not caspase‐11 is able to cleave IL‐1β and IL‐18.^20^, ^52^ However, both of these caspases are able to cleave gasdermin D and induce pyroptosis.^53^, ^54^ Hence, there must be biochemical differences that distinguish these closely related proteases. Using full length or CARD lacking recombinant expressed enzymes showed that the CARD domain does not inhibit the activity of caspase‐11 toward endogenous substrates. The authors predict that the specificity and activity of caspase‐11 would not be affected by the CARD domain. Until now, there has been no selective substrate for caspase‐11 over caspase‐1. This makes it difficult to design compounds that interfere with the non‐canonical pathway over the canonical one. Characterization of the putative exosite will pave the way for designing compounds that will selectively target caspase‐11.

## 4 IL‐1α IS A DIRECT SUBSTRATE OF MOUSE CASPASE‐11 AND HUMAN CASPASE‐5 DURING NON‐CANONICAL INFLAMMASOME ACTIVATION AND DURING SENESCENCE

Although IL‐1α and IL‐1β are released in response to non‐canonical inflammasome activation, the upstream target/s involved in cleaving and activating IL‐1α has/have not been characterized until recently. Wiggins et al showed that IL‐1α is specifically cleaved and activated by human caspase‐5 or mouse caspase‐11 at a conserved position adjacent to the calpain site.^55^ In human macrophages, caspase‐5 promotes cleaved IL‐1α release after inflammasome activation. In contrast to IL‐1β release that requires caspase‐11 and caspase‐1, the cleavage and release of IL‐1α is exclusively dependent upon caspase‐11 in murine macrophages. Importantly, IL‐1α acts in an autocrine/paracrine manner to promote senescence‐associated secretory phenotype (SASP),^56^, ^57^ where senescent cells develop altered secretory activities. SASP is involved in immune surveillance and clearance of senescent cells.^58^ Further, it is associated with the release of pro‐inflammatory cytokines, chemokines, proteases, and growth factors, thus driving chronic inflammation leading to diseases and unhealthy aging.^59^ Additionally, senescent human cells show an increase in caspase‐5 expression and total cleaved IL‐1α compared to growing cells. Knockdown of caspase‐5 reduces IL‐1α release and cytokines associated with SASP such as IL‐6, MCP1, and IL‐8, suggesting that caspase‐5 is required for IL‐1α release during senescence in vitro. We also showed that IL‐6, KC, and TNF‐α are drastically reduced in the absence of caspase‐11.^33^ Additionally, *caspase‐11^−/−^* mice showed reduction in the level of IL‐1α and KC in response to MRSA infection.^60^ Consequently, directly targeting caspase‐11/‐5 may reduce inflammation and limit the deleterious effects of senescent cells that accumulate during disease and aging.^55^

## 5 TRPC1 IS A SUBSTRATE FOR CASPASE‐11‐MEDIATED IL‐1β RELEASE

The cationic channel subunit transient receptor potential channel 1 (TRPC1) is a membrane protein that can form channel permeable to Ca^2+^. It was identified via yeast two‐hybrid screen and was shown to interact with the catalytic (P10 and P20), but not the CARD domain of caspase‐11. Co‐expression of the catalytic domains of caspase‐11, but not caspase‐1 with TRPC1 reduced the level of TRPC1 in HEK293 cells.^61^ Furthermore, incubation of purified recombinant caspase‐11 p30 with S^35^‐labeled TRPC1 in vitro led to appearance of multiple TRPC1 fragments and a reduction of full‐length TRPC1,^61^ suggesting that the TRPC1 is a substrate for caspase‐11. Since caspase‐11 is inducible via LPS, Py et al examined the effect of LPS treatment on the level of ectopically expressed TRPC1. It was shown that HA‐TRPC1 protein levels were significantly reduced, but not in the presence of the pan caspase inhibitor z‐VAD.fmk or IDUN‐6556, indicating that TRPC1 is degraded by inflammatory caspases. Moreover, TRPC1 degradation was shown in macrophages infected with *E coli* (J53 strain), or following prolonged stimulation with LPS (16hrs) followed by ATP stimulation. These stimulators have been shown to induce caspase‐11 expression. However, low dose of LPS stimulation followed by ATP, which is sufficient for caspase‐1 secretion but insufficient to induce the expression of caspase‐11, did not lead to TRPC1 degradation.^61^ Thus, caspase‐1 activation in the absence of caspase‐11 expression does not lead to TRPC1 degradation. In addition, caspase‐11‐mediated degradation of TRPC1 following LPS stimulation suggests that the TRPC1 is remodeled at the onset of inflammation and exhibits roles in regulating innate immunity.

Caspase‐11 has been shown to be required for caspase‐1 activation, IL‐1β and IL‐18 secretion, and pyroptosis in macrophages.^20^ Since caspase‐11 is a critical mediator of LPS‐induced septic shock, Py et al assessed the response of *trpc1^−/−^* mice to intraperitoneally injected LPS. Indeed, *trpc1^−/−^* mice showed higher IL‐1β in the serum following intraperitoneal LPS injection as compared to WT mice, suggesting that TRPC1 regulates inflammation in vivo via inhibiting IL‐1β secretion. Moreover, *trpc1^−/−^* macrophages showed higher secretion of mature IL‐1β and IL‐18 in response to *E coli*. However, TRPC1 deficiency did not have any effect on caspase‐1 cleavage, observed by appearance of the caspase‐1 subunit (p20) by Western blotting. Further, infection of *trpc1^−/−^* macrophages with *E coli* or treatment with canonical stimuli such as ATP did not sensitize cells to caspase‐11‐dependent pyroptosis, indicating that pyroptosis occurs independently of TRPC1 degradation. Additionally, *trpc1^−/−^* macrophages following LPS stimulation did not show an increase in pro‐IL‐1β, thus excluding the role for TRPC1 in TLR4 signaling. Therefore, caspase‐11 regulates IL‐1β secretion through degradation of TRPC1 and remodeling of associated channel complexes independently of caspase‐1.^61^ These data suggest that cleavage of TRPC1 likely impacts events downstream of caspase‐1 activation, including the unconventional secretion pathway that is responsible for mature IL‐1β release.^61^ The authors speculate that the inflammatory response may alter the gating properties of TRPC1‐containing channels in the plasma or intracellular membranes to promote unconventional protein secretion. Therefore, the possibility for the role of the TRPC1‐associated channelosome in regulating unconventional secretion needs further investigation.

## 6 INTRACELLULAR LPS, MODE OF CYTOSOLIC DELIVERY, AND DETECTION BY CASPASE‐11

Specific recognition of Gram‐negative bacteria via innate immune system requires activation of pathogen recognition receptors (PRRs) such as the TLRs, and intracellular receptors such as caspase‐11. The immense presence of bacterial LPS in the environment requires a tightly regulated detection and elimination of bacteria. Therefore, this necessitates the existence of multiple mechanisms for recognition and inflammatory‐based response to LPS. Intracellular contamination by LPS demands a strong response. Thus, an LPS sensor can mediate an exacerbated response, resulting in the release of inflammatory cytokines associated with pyroptosis. It was shown that caspase‐11 is activated by intracellular, live, and intact cytosolic LPS and the CARD domain of caspase‐11 is sufficient for binding to lipid A.^2^, ^28^ Moreover, maximum caspase‐11 activation is mediated by sensing the hexa‐acylated (lipid A with six acyl group), but not penta‐acylated (lipid A with five acyl group) or tetra‐acylated (lipid A with four acyl group) form of lipid A in the LPS structure.^2^, ^27^, ^62^ Therefore, bacteria that lacked intact LPS failed to activate caspase‐11.^2^, ^28^ However, modifying LPS structure by adopting penta‐acylated or tetra‐acylated LPS allows some pathogenic bacteria to evade the detection by caspase‐11 and to avoid provoking inflammation.^2^, ^31^

Since caspase‐11 was activated by non‐cytosolic and extracellular bacteria, the semantic question was how do Gram‐negative pathogens deliver their LPS to the cytosol to mediate activation of caspase‐11? Some bacteria such as the *Burkholderia* spp can access the cytosol following invasion of the cell, thus delivering their LPS to the cytosol.^28^ Other pathogens have secretion systems to deliver virulence factors into eukaryotic cells.^27^, ^63^ It has been demonstrated that clathrin‐mediated endocytosis of bacterial outer membrane vesicles (OMVs), which are overloaded with LPS, mediate the delivery of LPS into the host cell cytosol.^64^ Intriguingly, caspase‐11‐mediated activation, pyroptosis, and release of mature inflammatory cytokines were observed with purified OMVs derived from Gram‐negative but not Gram‐positive bacteria.^63^

Another mode of delivering cytosolic LPS was through a family of dynamin‐related large GTPases, the so‐called guanylate‐binding proteins (GBPs).^65^ Expression of GBPs is induced in response to interferons and other pro‐inflammatory cytokines.^66^ The GBPs promote a wide spectrum of innate immune functions against intracellular pathogens.^67^ Meunier and his colleagues showed that GBPs are required for activation of caspase‐11 in response to infection with vacuolar Gram‐negative bacteria.^65^ Indeed, induction of caspase‐11‐dependent pyroptosis by cytoplasmic *L pneumophila*‐derived LPS required GBPs.^68^ Further, macrophages lacking GBPs showed impaired caspase‐11 activation and attenuated pyroptosis.^68^ Recently, it has been shown that GBPs activate caspase‐11 and regulate non‐canonical NLRP3 inflammasome and IL‐1β release in response to T3SS‐negative *Pseudomonas (P). aeruginosa*.^69^ The fact that GBPs are exclusively relevant in inducing immune response to T3SS‐negative *P aeruginosa* demonstrates the that these mechanisms have evolved to detect pathogens that escape detection by canonical inflammasomes.^69^ Therefore, GBPs promote caspase‐11‐driven, cell‐autonomous immune defense against Gram‐negative pathogens accessing the cytosol.^68^ The cytosolic detection of intracellular LPS by caspase‐11 could serve as an extra checkpoint that is established proceeding to initiating irreversible steps in the cell.

## 7 GASDERMIN D, A SUBSTRATE FOR CASPASE‐11‐MEDIATED SECRETION OF IL‐1β AND IL‐18

In cases of high bacterial burdens, caspase‐11 mediates pyroptotic cell death, which consequently results in clearing intracellular bacteria.^6^, ^31^ Suicidal pyroptotic response inhibits the spread of some pathogens by destructing the cozy niche needed by replicating intracellular bacteria. Consequently, released bacteria become accessible to attack by extracellular neutrophils.^70^ For other organisms, pyroptosis actually helps propagation of the pathogen and the start of a new cycle of replication in a new cell.^71^ Furthermore, pyroptosis is considered a crucial defense mechanism against certain pathogens escaping phagosome‐lysosome fusion. Dysregulation or overactivation of pyroptosis results in excessive inflammation, which can lead to a number of inflammatory diseases and vital organ damage.^69^, ^70^

Pyroptosis is distinguished as canonical or non‐canonical depending on inflammatory caspases involved, which comprises caspase‐1 and caspase‐11/4/5, respectively. Although different inflammatory caspases can cause canonical or non‐canonical inflammasome‐mediated pyroptosis, some of them share a common substrate, gasdermin D.^53^, ^54^ Gasdermin D is highly expressed in the gastrointestinal epithelia and not restricted to macrophages, thus extending pyroptosis to other cell types.^74^ Similar to *caspase‐11^−/−^*, mice lacking gasdermin D are also protected from high dose of LPS‐mediated septic shock.^53^ Gasdermin D belongs to a large gasdermin family exhibiting a unique membrane pore‐forming activity. Full‐length gasdermin D has an N‐ and C‐terminal domains linked by a loop. The full‐length form is inactive due to the inhibitory effect of the C‐terminal on the N‐terminal domain.^53^, ^54^, ^75^ Cleavage of gasdermin D at an aspartate site within the linking loop by caspase‐1 or caspase‐11 removes the inhibitory effect by the C‐terminal domain, thus rendering it active.^54^ Similarly, the human caspase‐4/5 cleaves gasdermin D in response to cytosolic LPS treatment. Importantly, the N‐terminal domain of gasdermin D is competent and sufficient to promote pyroptosis, not only in vitro, but also in vivo.^53^, ^54^, ^76^ Moreover, the N‐terminal binds to negatively charged phosphoinositides and cardiolipin in the membrane, and lipid binding mediates oligomerization.^75^, ^77^ The oligomerized N‐terminal domain causes a pore, which dissipates the electrochemical gradient across the membrane and disrupts the osmotic potential, causing cell lysis. Gasdermin D‐deficient cells have impaired processing of caspase‐1 and release of IL‐1β in cell lysate following LPS electroporation.^78^ Further, gasdermin D pore indirectly activates the NLRP3 inflammasome to generate active caspase‐1, which cleaves pro‐IL‐1β to its mature form that is secreted.^79^ Plasma membrane pore formation by gasdermin D oligomers results in K^+^ efflux, which is an established trigger of NLRP3 activation.^80^, ^81^ This indicates that caspase‐11 activation of gasdermin D may precede NLRP3 activation of caspase‐1 and IL‐1β secretion. Several prominent studies have demonstrated that caspase‐11‐mediated functions, including the release of IL‐1β, are attributed to pyroptosis.^82^, ^83^

## 8 NON‐APOPTOTIC FUNCTIONS OF CASPASE‐11 AND GASDERMIN D

Recent study showed that at sublytic levels of inflammasome activation, and in the absence of pyroptotic cell death, gasdermin D pores mediate the release of IL‐1β or IL‐18 and other cytosolic proteins.^84^ Notably, we have shown that the release of IL‐1β from macrophages in response to MSU was not accompanied by cell death.^33^ Our findings are corroborated by recent reports describing how gasdermin forms pores within the intact plasma membrane, allowing the release of IL‐1β independently of cell death.^85^, ^86^ In a state of hyperactivation (truly viable phagocytes that release IL‐1), gasdermin D regulates IL‐1β release in these viable cells independent of cell lysis.^85^, ^86^ Using *Staphylococcus (S.) aureus OatA‐*deficient mutant (which did not induce the release of lactate dehydrogenase), it was shown that the extent of pore formation observed during *OatA‐*deficient infection was less than that observed for pyroptotic stimuli.^85^, ^86^ Furthermore, membrane pores formed by activated gasdermin D exhibit different sizes with variable stoichiometry.^84^ Therefore, the diameter and the size of the substrate dictate whether it is going to be released by the gasdermin D pore. Indeed, substrates with small diameter such as IL‐1β can pass through the gasdermin D pore. However, larger substrates such as the lactate dehydrogenase (LDH) require cell lysis.^84^ So gasdermin D pore exhibits a bifunctional role by mediating cell lysis‐dependent and cell lysis‐independent release of cytokines. Gasdermin D pores may act as unspecific channels that release cytosolic proteins in size‐dependent way, with a possible cutoff between 25 and 50 kDa.^84^ Interestingly, proteins released subsequently to inflammasome activation are secreted through the gasdermin D pore.^87^ These secreted proteins such as IL‐1α and HMBG1 are alarmins or DAMPs that alarm other cells, and therefore modulate the inflammatory responses downstream of inflammasome activation.^20^ The unconventional secretion of these endogenous alarmins, following cytosolic LPS recognition, is promoted by caspase‐11 and is independent of caspase‐1.^20^ The pore‐forming mechanism shared by the gasdermins could be utilized for future drug design for treating sepsis or other disease conditions characterized by inflammation. Together, gasdermin D exhibits distinct functions in the context of two different cell‐fate decisions. One function is to execute cell death, associated with indirect release of IL‐1 after membrane disruption, when the cell‐fate decision of pyroptosis is made. This cell‐fate decision is advantageous due to the massive inflammatory response that can be at the site of infection. However, pyroptosis is costly as the dead cell can no longer participate in any immunomodulatory activities. The second cell‐fate decision is mediated by gasdermin D and independent of cell death. The benefit of this cell‐fate decision is that the phagocyte can add IL‐1 family cytokines to the repertoire of secreted factors and that these cells might continue to influence immunomodulatory events.

## 9 PYROPTOTIC AND NON‐PYROPTOTIC ANTI‐BACTERIAL IMMUNE SURVEILLANCE ROLE OF CASPASE‐11

Given the extensive number of bacterial pathogens, it is expected that mammalian cells harbor several inflammasomes to sense wide arrays of pathogenic stimuli. Caspase‐11 is involved in protecting the host against a wide variety of extracellular and intracellular Gram‐negative pathogens.^6^, ^20^, ^27^, ^31^, ^88^, ^89^, ^90^ Indeed, wildtype mice clear cytosolic *Burkholderia (B.) thailandensis* and *B pseudomallei* and are resistant to the infection. However, *caspase‐11^−/−^* mice are highly susceptible to *B thailandensis* and *B pseudomallei* growth.^31^ Moreover, *Salmonella (S). Typhimurium* that exhibit vacuolar residence fail to activate caspase‐11, but instead activate the canonical inflammasome leading to IL‐1β release that is dependent upon NLRC4.^6^, ^31^ Intriguingly, a mutant strain of *S Typhimurium* (*ΔsifA*) is able to disrupt the *Salmonella* vacuolar membrane, thus gaining access to the cytosol and resulting in increased pyroptosis that is indispensable of caspase‐11.^31^ Therefore, *∆sifA* mutant is efficiently cleared by WT but not *caspase‐11^−/−^* mice. This suggests that WT vacuolar *S Typhimurium* evade the detection by caspase‐11‐mediated pyroptosis due to lack of access to the cytosol.^31^ Similarly, caspase‐11 restricts *L pneumophila ΔsdhA* mutant, which lyses the vacuole. This further indicates that caspase‐11 is required for clearing cytosolic bacteria.^31^ Intriguingly, caspase‐11‐mediated activation is dependent upon functional bacterial type III and IV secretion systems. Type III secretion mutant of *S Typhimurium* is defective in caspase‐4 activation, cell death, and release of IL‐1α and IL‐1β.^46^ Moreover, it has been demonstrated that functional type IV secretion is indispensable for caspase‐4‐ and caspase‐11‐dependent cell death during *Legionella (L.) pneumophila* infection of human macrophages.^46^, ^89^ It is considered that the needle apparatus of the secretion system damages the vacuolar membrane and allows leakage of inflammasome components across the vacuolar membrane into the cytosol.

**Figure 2:**
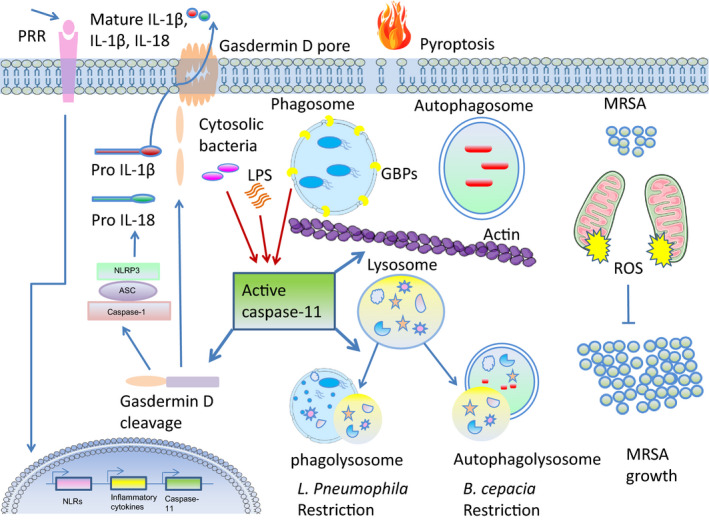
Caspase‐11 induction, activation, and downstream (pyroptotic and non‐pyroptotic) functions in modulating bacterial survival. Following the stimulation of cell surface pattern recognition receptors (PRRs), NOD‐like receptors (NLRs), inflammatory cytokines, and chemokines, and caspase‐11 is upregulated. Some vacuolar intracellular pathogens gain access to the cytosol via the action of guanylate‐binding proteins (GBPs). Upon lipopolysaccharide (LPS)‐mediated activation, caspase‐11 cleaves and activates gasdermin D, causing the inflammatory lytic cell death: pyroptosis. Downstream of the non‐canonical inflammasome, gasdermin D activates the canonical Nlrp3 inflammasome, promoting caspase‐1 activation, leading to maturation of IL‐1β and IL‐18 and pyroptosis. Strong activation of the inflammasome leads to release of cytokines and loss of membrane integrity, which results in release of damage chemicals to the extracellular space, thus alarming other immune cells. Caspase‐11 exhibits non‐pyroptotic functions that regulate bacterial load intracellularly. Caspase‐11 regulates vesicular trafficking by modulating actin cytoskeleton. It promotes fusion of phagosomes containing *L penumophila* with the lysosomes by regulating the ratio F/G actin through cofilin, thus restricting *Legionella pneumophila* replication in macrophages. Caspase‐11 also controls fusion of autophagosomes containing *B cepacia* fusion with the lysosome by regulating actin. For example, caspase‐11 counteracts mitochondrial ROS‐mediated clearance of *Staphylococcus aureus* in macrophages

Even though caspase‐11‐mediated pyroptosis restricts bacterial growth by demolishing the bacterial niche, other studies have demonstrated a non‐pyroptotic role of caspase‐11 in clearing bacteria.^30^, ^60^, ^91^, ^92^, ^93^ When used at a physiological MOI (0.025‐0.05), *L pneumophila* helps discern important physiological processes in the cell. However, when used at MOI of 10 or more, it becomes a great tool for the study of cell death. We and others using physiological levels of infection have shown that *caspase‐11^−/−^* macrophages support growth of *L pneumophila* as compared to WT macrophages.^30^ The uptake of *L pneumophila* was comparable in WT and *caspase‐11^−/−^* macrophages 1 hr postinfection.^30^ Additionally, the type IV secretion system Dot/Icm is required for intracellular *L pneumophila* replication and is modulated by caspase‐11. This was shown by the ability of both WT‐ and caspase‐11‐deficient macrophages to restrict the replication of *L pneumophila dotA^−/−^* mutant.^30^ Confirmation of the role of caspase‐11 in restricting *L pneumophila* growth in macrophages was demonstrated through complementation (by a plasmid expressing caspase‐4 and caspase‐5) or depletion of caspase‐11 by siRNA. These series of experiments demonstrated that ectopic expression of both caspase‐4 and caspase‐5 in human THP1 macrophages, which normally allow *L pneumophila* growth, restricts *L pneumophila* growth. Depletion of these caspases on the other hand mediated growth of *L pneumophila* in WT mouse macrophages, which are usually restrictive.^30^ Mechanistically, caspase‐11 promotes fusion of the phagosomes containing *L pneumophila* with the lysosomes, thus restricting *L pneumophila* replication in WT macrophages.^30^ Figure 2 shows non‐pyroptotic functions of caspase‐11 in the context of bacterial infection. It is important to note that most of the studies described above were obtained from mice in C57BL/6 background. Other important studies in mixed background like that of Zamboni's group concluded that caspase‐11 does not restrict *L pneumophila* infection in the 129 × C57BL/6 background. Therefore, the interpretation of caspase‐11 functions should also take into consideration the genetic composition of the mouse model used.^94^

Further, caspase‐11 positively modulates autophagosome formation and trafficking to lysosomes in murine macrophages infected with *Burkholderia cenocepacia*.^90^ Intriguingly, Thurston et al showed that a subpopulation of cells harboring cytosolic bacteria did not undergo cell lysis, but, instead, caspase‐11‐mediated restriction preceded the onset of cell death and was independent on gasdermin D and IL‐1β.^92^ Together, these data suggest that caspase‐11 exhibits additional effector mechanism besides its typical pyroptotic function to control intracellular infections.

The action of inflammasome is not restricted to myeloid cells, caspase‐11 is expressed constitutively at high basal level in colonic mucosa.^95^ Importantly, the intestinal epithelial cells (IECs) employ both the canonical and non‐canonical inflammasomes to initiate mucosal defense against enteric pathogens.^96^, ^97^, ^98^ C*aspase‐11^−/−^* mice harbor higher *S Typhimurium* in the IECs,^96^ which is required for IL‐18 secretion and *S Typhimurium* clearance. Further, reduced cell death following infection with *S Typhimurium*,* Enteropathogenic E coli*, and *Shigella (S). flexneri* was observed in human colonic epithelial cells following siRNA knockdown of caspase‐4.^96^, ^99^ Intriguingly, bacterial effector proteins can inhibit caspase‐4 activation. For example, the *Enteropathogenic E coli* NleF and the *S flexneri* OspC3 type III effector proteins inhibit caspase‐4 activation and proteolytic‐mediated function.^99^ Kobayashi et al showed that *S flexneri* deposits conserved OspC3 X1‐Y‐X2‐D‐X3 motif at the putative catalytic pocket of caspase‐4, preventing caspase‐4‐p19 and caspase‐4‐p10 heterodimerization.^99^ Collectively, caspase‐11 represents auxiliary defense mechanisms that destabilize and subsequently rupture bacterial‐containing vacuole, thus exposing the vacuolar‐resident pathogen to the inflammasome.

Little is known about the roles of caspase‐11 in the immune defense against Gram‐positive bacteria. Earlier reports indicated that caspase‐11 has no role in protection against *Listeria monocytogenes* infection.^100^ Recently, cytosolic lipoteichoic acid (LTA), a molecule produced by Gram‐positive bacteria, or infection with *L monocytogenes*, was reported to induce caspase‐11 activity via NLRP6.^101^ However, unlike mice infected with Gram‐negative bacteria, *caspase‐11^−/−^* mice exhibit improved survival along with efficient bacterial clearance of Gram‐positive pathogens such as *L monocytogenes* and *S aureus*.^101^ Thus indicating that the absence of caspase‐11 helps with the clearance of some Gram‐positive bacteria. Using three different multiplicity of infections (MOIs), our group showed that *caspase‐11^−/−^* macrophages promoted efficient clearance of methicillin‐resistant *S aureus* (MRSA) with increasing MOI. Further, lungs of *caspase‐11^−/−^* mice exhibit reduced MRSA as compared to WT mice.^60^ Thus, the in vivo and in vitro data suggest that the macrophage response against MRSA is more effective in the absence of caspase‐11. The improved MRSA clearance seen in *caspase‐11^−/−^* macrophages was independent of cell death. Instead, *caspase‐11^−/−^* macrophages was shown to have an increased association of MRSA with mitochondria. Moreover, electron microscopy revealed more bacteria in proximity to mitochondria in *caspase‐11^−/−^*. Furthermore, measurement of mitochondrial superoxide production, using the MitoSOX Red reagent, showed that MRSA suppressed superoxide levels in WT macrophages. Notably, macrophages lacking caspase‐11 exhibited higher MitoSOX fluorescence in response to MRSA than corresponding WT cells.^60^ In addition, pharmacological inhibition of mitochondrial complex III in the electron transport chain (ETC) by antimycin A (Ant‐A) treatment, which raises mitochondrial superoxide production, augments the bactericidal capacity of *caspase‐11^−/−^* macrophages against MRSA.^60^ This suggests that the enhanced proximity to mitochondria contributes to MRSA elimination in response to Ant‐A‐induced mitochondrial superoxide generation in *caspase‐11^−/−^* macrophages. Intriguingly, increased association of MRSA with the mitochondria in *caspase‐11^−/−^* macrophages was shown to be associated with impaired actin dynamics. Thus, inhibition of actin cytoskeleton via cytochalasin D (Cyto D) prevents the dissociation of phagocytosed MRSA from mitochondria, and hence restores Ant‐A‐induced bacterial killing in WT macrophages. Together, our study provides a novel role for caspase‐11 in the persistence of Gram‐positive bacteria by modulating the actin machinery.

## 10 ROLE OF CASPASE‐11 IN MODULATING INTRACELLULAR TRAFFICKING

Vesicular trafficking and fusion of the phagosome‐containing pathogen with the lysosome is a central host immune mechanism modulated by the actin machinery. In order for phagolysosomal fusion to happen, actin nucleates and polymerizes around the membranes of phagosomes allowing a directional track to interact and mediates fusion with lysosomes.^102^, ^103^ The significance of caspase‐11 during the life cycle of *L pneumophila* in macrophages was shown by modulating trafficking of *L pneumophila*‐containing vacuole (LCV) with the lysosomes and actin dynamics.^30^ Trafficking of the LCV is halted, and *L pneumophila* evades lysosomal degradation in murine macrophages deficient of caspase‐11.^30^ Importantly, caspase‐11 functional activity is required for promoting its function, as macrophages expressing the WT, but not the inactive mutant caspase‐11, mediated fusion of phagosomes containing *L pneumophila* with the lysosomes.^30^, ^91^ Indeed, mouse macrophages show low amount of polymerized actin in the vicinity of phagosomes containing *L pneumophila* in *caspase‐11^−/−^* macrophages, which is a result of modulating the phosphorylation status of cofilin via targeting RhoGTPase.^30^, ^91^ Lack of caspase‐11 maintained cofilin in the unphosphorylated active form, thus sustaining actin depolymerization, which hinders proper phagosome‐lysosome fusion.^91^ Furthermore, transportation of phagosomes containing non‐pathogenic *E coli* is not influenced by caspase‐11, as vacuoles containing *E coli* are destined to the lysosome irrespective of caspase‐11.^88^, ^91^ These data suggest differential regulation of the endocytic pathways by caspase‐11.^30^

Together, caspase‐11 regulates actin polymerization through cofilin, affecting subcellular organization of vesicles and organelles without affecting phagocytic events.^30^, ^91^ Therefore, the balance in cofilin activation is fundamental in promoting actin polymerization and efficient phagolysosomal fusion required for destruction of the intracellular pathogen.^30^, ^91^ These data shed the light on the role of caspase‐11 in modulating the actin dynamics and how an intracellular pathogen could manipulate these dynamics to promote survival intracellularly by evading vesicular trafficking. This further reveals that immune cells activate caspase‐11 to mediate cellular immunity by using an alternative non‐apoptotic mechanism to restrict bacterial replication.^92^

## 11 ROLE OF CASPASE‐11 IN MODULATING AUTOPHAGY IN RESPONSE TO BACTERIAL INFECTION

Autophagy, an intracellular catabolic pathway, is important for cellular homeostasis and recycling of damaged organelles and proteins, as well as clearance of intracellular pathogens.^104^ Typical stimuli known to induce autophagy are nutrient deprivation, increased production of reactive oxygen species, and the accumulation of protein aggregates. The breakdown of damaged organelles and proteins via autophagy leads to recycling of nutrients and sustains cellular energy levels. In addition, several intracellular bacteria are cleared by a bacterial‐specific autophagy, termed xenophagy.^105^, ^106^, ^107^, ^108^, ^109^

*Legionella pneumophila* is a great tool to understand autophagy; however, its behavior varies according to the MOI and to the cell type and mouse background. In primary C57BL/6 macrophages, non‐opsonized *L pneumophila* is degraded by autophagy. This process requires the expression of a *L pneumophila* factor called LegA9.^110^ However, in HEK293T cells, opsonized WT *L pneumophila* was able to avoid autophagy via its effector RavZ. On the other hand, studies in WT murine macrophages used *fla* mutants to dissect the role of RavZ in autophagy.^111^ Therefore, it is important, especially in the *L pneumophila* field, to take into account the mouse background, the type of cells, and whether the organism is opsonized before comparing and contrasting the conclusions of different studies.

During *L pneumophila* infection, inflammasome activation promotes autophagy.^112^ Similarly, caspase‐11 positively modulates autophagosome formation and trafficking to lysosomes in murine macrophages infected with *B cenocepacia*.^90^ Lungs of *caspase‐11^−/−^* mice and their derived macrophages increased *B cenocepacia* colony‐forming units (CFUs). The permissiveness of lungs or macrophages derived from *caspase‐11^−/−^* mice to *B cenocepacia* is associated with defect in autophagosome maturation during infection.^90^ This is accompanied by compromised transport to lysosomes resulting in the accumulation of Rab7 and decreased colocalization with LC3, a marker that has been used to denote autophagy.^90^ Importantly, dysfunctional regulation of actin dynamics via cofilin‐1 in *caspase‐11^−/−^* compromises the formation and trafficking of autophagosomes leading to accumulation of Rab7 during *B cenocepacia* infection. *Burkholderia cenocepacia* infection in *caspase‐11^−/−^* mice and their derived macrophages produced less cytokines (IL‐1α, IL‐1β) and chemokines (CXCL1/KC) compared to WT in their BALF. Therefore, the increased survival of *caspase‐11^−/−^* mice is associated with decreased inflammation shown by less cytokines and chemokines produced in response to infection with *B cenocepacia*.^90^ Other study demonstrated that the *Salmonella*‐containing vacuole (SCV) is recognized by GBPs, which mediate the lysis of the SCV resulting in the release of the bacteria into the cytosol and activation of caspase‐11 by bacterial LPS. In addition, lysed vacuoles are followed by recruitment of the autophagy machinery. Thus, uptake of the bacterium and the lysed vacuole into autophagosomes reduces caspase‐11 activation by removing the source of LPS from the cytosol.^65^ Although it is not known how autophagy regulates caspase‐11 activation, it is possible that autophagy removes excessive reactive oxygen species that are suggested to enhance caspase‐11 expression.^113^ Autophagic removal of intracellular DAMPs, inflammasome components, or cytokines can reduce inflammasome activation. Similarly, inflammasomes can regulate the autophagic process, allowing for a two‐way mutual regulation of inflammation that may hold the key for treatment of multiple diseases. Therefore, mutual regulation of caspase‐11 activation and autophagy is important for the maintenance of cellular homeostasis and optimized innate immune response to intracellular bacterial pathogens.

## 12 ROLE OF CASPASE‐11 IN CELL MIGRATION

It has been demonstrated that caspase‐11 facilitates cell migration of different cell types during inflammation. Intriguingly, splenocytes and macrophages derived from *caspase‐11^−/−^* mice are defective in migration toward different chemokines in vitro and in vivo.^114^ In vitro actin depolymerization assays demonstrated that caspase‐11 interacts physically and functionally with actin‐interacting protein 1 (Aip1).^114^ The resulting interaction increases the proximity of Aip1 to cofilin and F‐actin and facilitates cofilin‐mediated actin depolymerization. Moreover, *caspase‐11^−/−^* T cells migrate less efficiently into lymphoid tissues.^115^ Thus, caspase‐11 expression may regulate migration to the site of infection of cells that are implicated in early immune response and cytokine release. In addition, modulation of actin polymerization by caspase‐11 could regulate additional aspects of T‐cell biology, including T‐cell receptor (TCR) signaling.^116^ Since caspase‐11 positively regulates actin depolymerization leading to an increase in F‐actin in *caspase‐11^−/−^* cells, phalloidin staining was augmented in *caspase‐11^−/−^* compared to WT T cells after stimulation with low‐affinity ligand.^116^ Therefore, caspase‐11 regulates actin polymerization, which affects many cell functions.^116^

Studies in our laboratory have shown that the lack of caspase‐11 is associated with significant reduction in neutrophil influx and in the levels of inflammatory cytokines in the synovial fluid of MSU‐injected joints. Moreover, *caspase‐11^−/−^* mice injected with MSU crystals showed significant reduction in signs of joint inflammation. This was apparent by reduction in infiltration of inflammatory cells and swelling and tissue damage in the tibiotarsal joint space in *caspase‐11^−/−^* mice.^33^ Further, *caspase‐11^−/−^* mice showed reduced KC (IL‐8) production, a key player in neutrophil chemotaxis. Intriguingly, the absence of caspase‐11 is accompanied by reduced macrophage and neutrophil migration toward exogenously injected KC in vivo and in vitro.^33^ Therefore, the low cytokine production within the MSU‐injected joint and the innate changes in the ability of *caspase‐11^−/−^* neutrophils to migrate toward a chemotactic signal are correlated with the reduced neutrophil infiltration.^33^ In contrast to WT, *caspase‐11 ^−/−^* neutrophils exhibited irregular migration in response to a KC gradient in vitro. Intriguingly, we have found that in response to MSU, *caspase‐11 ^−/−^* neutrophils showed significantly less neutrophil extracellular traps (NETs). Reduced NET formation was independent of the receptor‐interacting protein kinase 3 (RIPK3), mixed lineage kinase domain‐like (MLKL) pathway, and gasdermin D. However, in *caspase‐11^−/−^* neutrophils showed altered cofilin phosphorylation in response to MSU. Together, our data further emphasize the non‐apoptotic functions for caspase‐11 in macrophages and neutrophils.

## 13 ROLE OF CASPASE‐11 IN ALLERGY AND ASTHMA

Importantly, caspase‐4/11 contributes to allergic airway inflammation, with implications for pathophysiology of asthma. Two independent studies have shown that caspase‐11‐deficient mice are resistant to developing experimental allergic airway inflammation in response to two different allergens. We used the common aeroallergen house dust mites (HDM), which has been demonstrated to induce sensitization in 85% of patients with asthma.^117^ Our data show a global reduction in inflammation in the lungs of *caspase‐11^−/−^*. This reduction is manifested by reduced cellular infiltration including neutrophils, macrophages, and lymphocytes in the lungs. We also observed reduced Th1, Th2, and Th17 cytokines in the broncho‐alveolar lavage fluid derived from *caspase‐11^−/−^* mice (Abu Khweek et al under review). The reduced lung inflammation in *caspase‐11^−/−^* could be due to increased level of IgA, which potentially neutralizes HDM in our experimental model. It can also be due to reduced migration of neutrophils to the lungs in response to lower KC and IL‐17A. Alternatively, it may be due to reduced IL‐33 that can lead to reduced IL‐4 and IL‐5 production by T cells. Our study offers several intriguing scenarios for the diverse functions of caspase‐11. Expression of caspase‐11 in innate immune cells following HDM exposure may be required for the antigen presentation by macrophages and dendritic cells to naive T cells. Subsequently, insufficient antigen presentation leads to reduction in cytokines and chemokines released in the BAL fluids. Our published work and that of others also support the notion for inherent defect in migration of *caspase‐11^−/−^* cells due to defect in the actin cytoskeleton.^33^, ^114^ Zaslona et al showed that *caspase‐11^−/−^* mice injected and challenged with ova plus alum are resistant to developing allergic airway inflammation. The reduced inflammation is associated with lessened infiltration of leukocytes to the lungs, especially eosinophils. In addition, *caspase‐11^−/−^* mice showed reduced release of Th2, Th1, Th17 cytokines, and circulating IgE.^118^ Even though we used a different allergen, exposure time, and routes of administration, the Zaslona findings are in line with several of our data. These studies demonstrate that caspase‐11 plays a protective role in asthma.

## 14 CONCLUSIONS

Different studies indicate that the inflammatory caspases have functions beyond those associated with pyroptosis and cytokine release. The canonical inflammasome has been studied extensively including stimuli, activation, and downstream effector functions. However, research regarding stimuli, activation mode, downstream substrates, and effector functions of the non‐canonical inflammasome are still in infancy. Given the multitude of signaling pathways and transcription factors that can interact with caspase‐11 promoter region, it is hard to associate a single PAMPs or DAMPs with the main transcription factor that is responsible for induction of caspase‐11 expression. The vital role of caspase‐11 in various cellular processes including inflammation supports the notion of tight regulation of this caspase in cases of stress or infection. Caspase‐11 is inducible, and its expression may serve to deliver cytokine‐producing cells to the site of infection in a rapid fashion, whereas apoptosis regulated by caspase‐11 may function as an auto‐regulatory mechanism to terminate the cytokine release.

Several studies have shed the light on the role of caspase‐11 in controlling bacterial infection, specifically how intracellular, extracellular, or vacuolar Gram‐negative bacteria access the cytosol, and their derived LPS mediated caspase‐11 downstream functions. It was shown that caspase‐11 is necessary for clearing cytosolic bacteria or pathogens that evade detection by the canonical inflammasomes. These studies showed that caspase‐11 operates by mediating pyroptotic and non‐pyroptotic functions to contain bacterial functions. Recent studies showed that caspase‐11 modulates the actin cytoskeletal machinery. Actin dynamics are involved in regulating multiple cellular events including cell migration, cell division, and secretion. The ability of caspase‐11 to regulate actin depolymerization may provide a potential mechanism for caspase‐11‐mediated control of additional events during inflammatory responses.

Despite these important advances, several gaps need to be unraveled to increase our understanding of caspase‐11‐mediated functions. These include (a) the signals that activate caspase‐11; (b) why only pathogenic bacteria mediate caspase‐11 activation; and (c) how caspase‐11 mediates caspase‐1 activation. The stoichiometry of the caspase‐11‐LPS complex is not defined, and it remains unclear whether the higher order caspase‐4/5/11 structures induced by LPS are true oligomers, or represent multiple caspase dimers binding to single LPS molecule or LPS aggregate. Understanding the diverse functions of caspase‐11 will pave the way for designing therapeutics.

## CONFLICT OF INTEREST

The authors of the manuscript declare that the submitted work was carried out in the absence of any personal, professional, or financial relationships that could potentially be construed as a conflict of interest.

## AUTHOR CONTRIBUTIONS

AAK wrote the review. AOA edited the manuscript.
